# Prevalence and associated factors of coexisting forms of malnutrition in children under 5 years age in a rural area of Cameroon

**DOI:** 10.1371/journal.pone.0303611

**Published:** 2024-06-10

**Authors:** Boris Ronald Tonou Tchuente, Maxwell Wandji Nguedjo, Dany Joël Ngassa Ngoumen, Grace Cynthia Fandio De Wandji, Hippolyte Tene Mouafo, Betrand Ayuk Tambe, Gabriel Nama Medoua, Vigny Ndomo Tsamo

**Affiliations:** 1 Centre for Food, Food Security and Nutrition Research, Institute of Medical Research and Medicinal Plant Studies, Yaounde, Cameroon; 2 Department of Biochemistry, Faculty of Sciences, University of Yaounde, Yaounde, Cameroon; 3 Nkong-Ni Subdivisionnnal Medical Center, Menoua Division, West Region of Cameroon, Dschang, Cameroon; 4 Department of Public Health and Hygiene, Faculty of Health Sciences, University of Buea, Buea, Cameroon; Federal University of Agriculture Abeokuta, NIGERIA

## Abstract

**Background:**

Malnutrition of children under 5 years of age is persistent in Cameroon principally in rural areas. Moreover, there is limited knowledge of coexisting forms of malnutrition (CFM) among children of this age. Therefore, the aim of this study was to assess the prevalence of CFM in a cohort of children under 5 years and to identify the associated factors.

**Methods:**

A cross-sectional study was conducted in the Health Districts of the locality of Dschang in the West region of Cameroon between June 2021 to November 2021. Data were collected from 200 under-five children of both sexes and an interviewer-administered questionnaire was administered to consented children’s mothers/guardians. Malnutrition in children was assessed by WHO growth standards (weight-for-height, weight-for-age, height-for-age and body mass index-for-age). The different CFM were defined by the presence of two autonomous forms of malnutrition in the same child. Logistic regression analyses were done to identify factors associated to different coexisting forms of malnutrition.

**Results:**

The results obtained showed prevalences of 4.20% for the coexistence of underweight with wasting, 7.8% for the coexistence of underweight with stunting and 14.8% for the coexistence of stunting with overweight. Lower maternal age (15–24 years old; OR = 0.09; p = 0.05) and lower education level (primary education, OR = 23.33; p = 0.00) were associated with the coexistence of underweight with wasting. Marital status (single mother, OR = 0.28; p = 0.00) was associated to the coexistence of stunting with overweight/obesity.

**Conclusion:**

The findings of this study provide evidence on the coexistence of different forms of malnutrition among children below five years of age in rural area of Cameroon. These finding would guide future research, policies, and programs on the management of malnutrition in rural areas of Cameroon.

## Introduction

With the current figure of malnutrition among children below five years, the world is not in good line to achieve the Sustainable Development Goals required to eliminate malnutrition [1]. Malnutrition can be categorized in 3 different forms: undernutrition (Stunting, wasting, and underweight), overnutrition (overweight or obesity), and micronutrient-related malnutrition (vitamin and mineral deficiency) [2]. It has been reported that 21.9%, 7.3% and 5.9% of children below five are respectively stunted, wasted and overweight. Africa is one of the most affected regions representing more than one-third of all stunted children and almost one-quarter of wasted and overweight/obese children [3].

There are cases where children suffer from more than one form of malnutrition viewed as coexisting forms of malnutrition (CFM) [4, 5]. Different combinations of CFM have been found including the combination of stunting with overweight/obesity, the combination of underweight with wasting or with stunting [6] and coexistence of stunting with overweight/obesity [7]. The presence of a single form (stunting or wasting) in an infant or child is well known to be associated with negative health outcomes such as impairment of cognitive development, child’s growth and immune system leading to an increased risk of morbidity and mortality [8].

When there is CFM in children, the gravity of health outcomes and the risk of death are greater compared to children with single forms of malnutrition [9, 10]. Furthermore, it is profoundly complicated to manage and prevent coexisting forms of malnutrition than a single form of malnutrition. When coexisting nutritional disorders are opposite such as coexistence of obesity with stunting, the management of both combinate form need to deal with nutritional cause related to each specific form [11, 12]. Although, its importance in terms of comorbidity and mortality, there are presently no joint global or regional estimates for CFM [2]. CFM was only assessed in a few geographical regions of the world [7, 13].

In Cameroon, malnutrition of children under five years is persistent principally in rural areas [14]. However, we know very little or not about the prevalence and contributing factors of CFM. This potentially limiting the knowledge on causes and consequences of child’s malnutrition which represents one of the leading causes of children death [15, 16]. In that regard, to better prevent child’s malnutrition and make effective policies there is a need for more research about it at communal, regional or national level. Thus, this study aimed to assess the prevalence of CFM in a cohort of children under five years, and to identify the factors associated with this coexisting form of malnutrition.

## Materials and methods

### Study area and period

A cross-sectional study was conducted in four medical centres of Nkong-Ni (Menoua division, West region, Cameroon): the Nkong-Ni district medical centre, the Saint Kisito health centre, the Misericorde health centre and the Mebou health centre. The study was carried out once a week, corresponding to the children’s vaccination day in each healthcare establishment. This study began on 30^th^ June 2021 and ended on 27^th^ November.

### Inclusion and exclusion criteria

Only children aged between 0–5 years whose mother or legal representative gave consent were included in the study. Besides, those with a health problem (fever, malaria, and diarrhea) likely to distort anthropometric measurements were excluded.

### Sample size determination

Children under the age of five and their mothers were randomly recruited during health campaigns on nutrition organized during vaccination days in health centres. The sample size was determined using the formula opposite:

$$N=\frac{t^{2}\times p(1-p)}{m^{2}}$$

N = required sample size

t = confidence level at 95% (standard value of 1.96)

p = prevalence of malnutrition in Cameroonian children aged 0 to 5 years is 11% [17].

m = margin of error at 5% (standard value of 0.05)

$$N=\frac{{1,96}^{2}\times 0,11(1-0,11)}{{0,05}^{2}}=150$$

**Fig 1:**
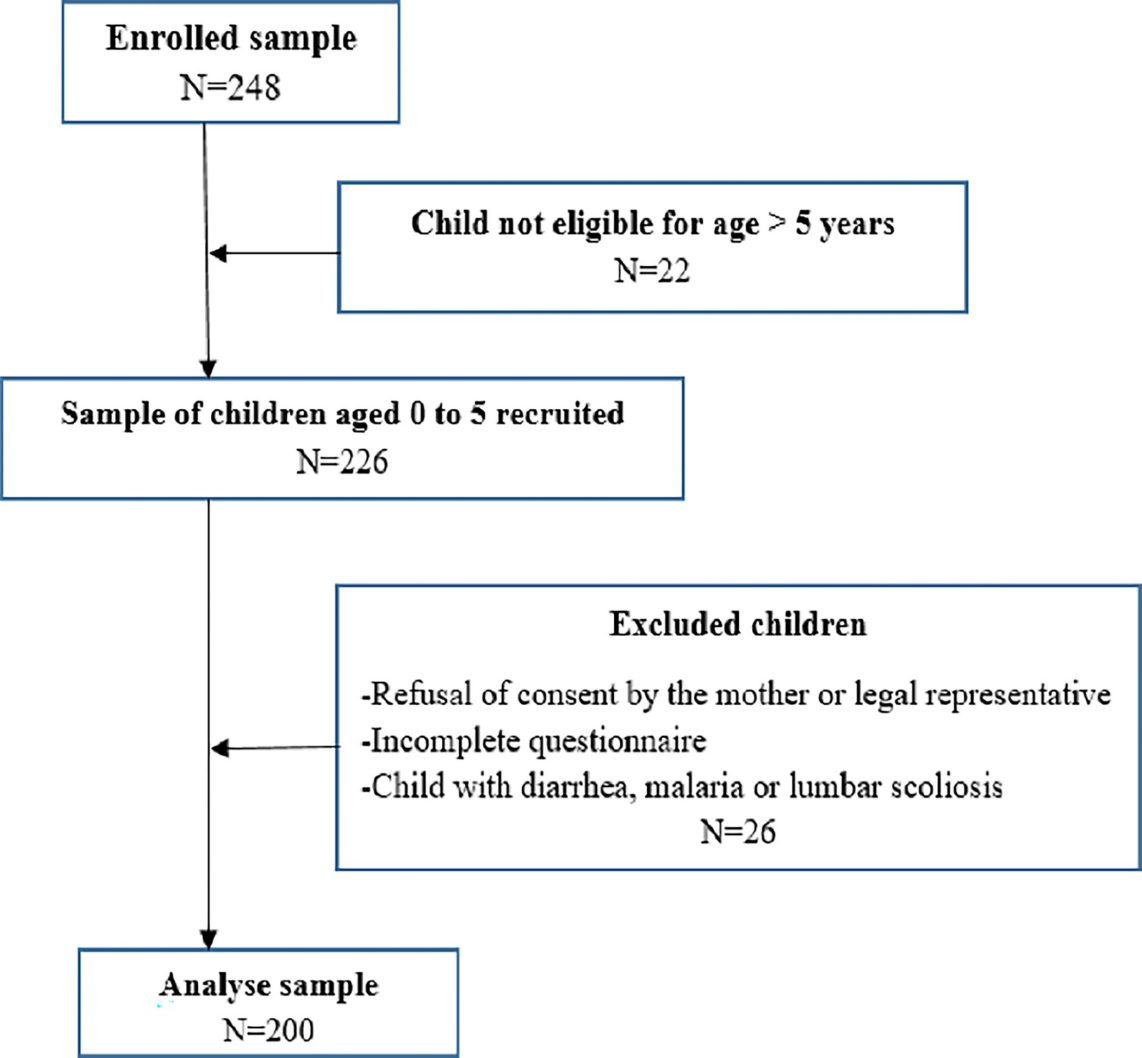
Flow chart of sample selection. N refers to number of participant for each category.

Taking into account the 10% non-response rate, the sample size is: N = 150 + 15 = 165 children. Therefore, at least 165 children are required for the study. After sampling, 200 children were selected for the study (Fig 1).

### Data collection

Before the field survey, a structured questionnaire written in French or English was pre-tested with the children’s mothers to ensure its reliability and validity. The interviewers were trained to administer the questionnaire. During the training of the interviewers, we studied the content of the questionnaire, a sales pitch to present the survey to the mothers to be interviewed, as well as the understanding and translation of specific terms, to limit the biases linked to improvised translation.

During the survey, data was collected on vaccination days in each of the health centres. The nurses were responsible for taking anthropometric and clinical parameters from the children, while the trained interviewers were responsible for administering the questionnaire to the mothers.

### Administration of the questionnaire

The questionnaire was administered to the mothers after they had given their free and informed consent. The questionnaire was administered via a face-to-face interview. It was used to obtain the mother’s socio-demographic (marital status, level of education) and socio-economic (source of income, occupation and socio-economic status) information, to identify the child, and data on the household’s eating habits and food security.

### Socio-economic status

The socio-economic level of the mothers was assessed using a composite index that took into account ownership of a bicycle, motorbike, car, television, radio, refrigerator; electricity, water in the house; material of the walls and floor; type of fuel used for cooking; status of the household in the dwelling occupied (owner, tenant, etc.). The first eight items were dichotomous and coded "1" if the property was present and "0" if it was absent. The next two items were also dichotomous and coded "1" for a cement wall or floor, and "0" for any other material. The fuel used for cooking was coded as 1; 0.5 and 0 for oil or gas, charcoal and firewood, respectively. Household status in the occupied dwelling was also dichotomous and coded "1" if "owner" and "0" for any other status. The amenity score (or SES) was the sum of these items up to a maximum of 12. On the basis of score tertiles, three levels were identified: low (0 to 2), medium (3 to 4) and high (> 5) [18].

### Assessment of food security status

Food security was assessed using the Household Food Insecurity Access Scale (HFIAS) questionnaire. There are 9 items in this questionnaire. For each item of the questionnaire, respondents indicated whether they had a particular experience of food insecurity in the four weeks prior to the survey and their exposure. The score is calculated by assigning a code ’0’ to all cases where the answer to the corresponding question is ’No’ and a code ’1’ to all cases where the answer to the question is ’Yes’. When the answer to the question was ’yes’, a score of ’’1’’, ’’2’’ or ’’3’’ was assigned respectively to the frequencies of occurrence ’rarely’, ’sometimes’ or ’often’. The score was obtained by summing the different ratings. The scores are obtained by adding together the different ratings. The highest score was 27 (when the answer to all nine frequency-of-occurrence questions was "often," coded as "3") and the lowest score was 0, when the participant answered "No" to all the questions. The higher the score, the more food insecure the participant; the lower the score, the less food insecure the participant [19]. Thus, when the HFIAS is 0, households are food secure; when the HFIAS is 0, households are food secure; when the HFIAS is between 1 and 8, households are mildly food insecure; when the HFIAS is between 9 and 13, household food insecurity is moderate; when the HFIAS is between 14 and 27, household food insecurity is severe.

### Anthropometric indices

Anthropometric data were collected by measuring weight and height. These were measured according to standard procedures as defined by the WHO, [20]. Height was estimated in centimeters using a SECA 416 infantiometer. The length of the child under 23 months of age was taken without the clothes, lying down with feet together on the infantiometer and the distance between the top of the head and the soles of the feet was measured. Children over 23 months of age stood with their feet together on the infantiometer and the distance between the top of their head and the soles of their feet was measured. Weight was measured in kilograms using a SECA 354 electronic baby scale. Both the height/length and the weight of children were measured twice and the average was taken.

### Diagnosis of malnutrition in children

Z-scores for weight-for-height, weight-for-age, height-for-age and BMI-for-age were calculated using WHO Anthro version 3.2.2 software to assess malnutrition in children.

➢ **Weight-for–Height: Z-score (WHZ)**

Acute malnutrition or wasting was assessed using the Z-scores of weight for height (**WHZ**): severe acute malnutrition (SAM) considered from a Z-score below -3 SD; moderate acute malnutrition (MAM) expressed from Z-scores between -3 SD and -2 SD, normal nutritional status (NNS) expressed from Z-score values greater than or equal to -2 SD [21].

➢ **Height-for-Age: Z-score (HAZ)**

Chronic malnutrition or stunting expressed from height-for-age (**HAZ**) Z-score values. Thus, severe chronic malnutrition (SCM) is observed from a Z-score below -3 SD; moderate chronic malnutrition (MCM) is expressed from Z-scores between -3 and -2 SD, normal nutritional status (NNS) is characterized by Z-score values greater than or equal to -2 SD [21].

➢ **Weight-for-Age: Z-scores (WAZ)**

Weight change was characterised by weight-for-age (WAZ) Z-score values. Underweight (WAZ) was identified by a Z-score less than -3 SD; normal weight status (NWS) was identified by Z-scores greater than or equal to -2 SD [21].

➢ **BMI -for -Age: Z-scores (BAZ)**

Overweight/obesity was assessed using BMI-for-age (BAZ) Z-scores as recommended by the WHO for children aged 0–5 years. B/A Z-score values < -2, > 1, > 2, and > 3 were used to diagnose children who were thin, at risk of being overweight, overweight and obese respectively [22].

### Identification of coexisting forms of malnutrition

The presence in the same child of two autonomous forms of malnutrition defined the CFM. Based on the nutritional status of each child, 3 categories of coexisting forms of malnutrition were considered: underweight with stunting, underweight with wasting, and stunting with overweight/obesity [7].

### Quality control

The equipment used were infantiometer and electronic baby scale. To ensure the quality of data generated by these equipment, there were calibrated daily. In addition, the investigators had undergone specific and rigorous training in the use of the measuring equipment. They also passed an examination prior to the survey, and only the best candidates were selected.

### Data management and statistical analysis

The data obtained in each centre were entered as they were collected, coded and entered into Microsoft Excel version 16, then exported to SPSS version 25.0 for analysis. WHO anthro plus was used for calculation of Z-scores from the anthropometric measurements. Descriptive statistics were used to analyse the socio-demographic data. The data were expressed as frequencies. The Pearson chi-square test was used to compare proportions between categorical variables. Unadjusted bivariate logistic regression analyses were performed to identify each risk factor associated with the different CFM in the study population. These analyses were used to obtain estimates of the Odd ratios and their respective confidence intervals. Variables with p-values <0.05 were considered to be statistically significant.

### Ethical consideration

The study was conducted in strict compliance with medical ethics, in accordance with the principles described in the Declaration of Helsinki. All the mothers of children aged 0–5 gave their free, written and informed consent for them and their child to take part in the study. A research proposal was submitted to the Institutional Review Board of the University of Buea and the ethical clearance was obtained (No: 2021/1467 04/UB/SG/IRB/FHS).

## Results

### Characteristics of study population

Table 1 shows characteristics of mothers and children. Forty-four percent of the mothers were aged between 15 and 24 years. The majority of mothers had studied up to secondary school (58.10%) and were living as a couple (67.20%). Twenty-six percent of mothers had source of income from trade and most of them were vendor/market seller (31.30%). Most of mothers had high socio-economic level (61.40%) and were food secure (61.60%). Ninety-two percent (92.00%) of children were younger than eleven months and majority of them were girls (51.20%).

**Table 1:** Characteristics of mothers and children.

Variables	Frequency	Percentage (%)
**Socio-demographics characteristic of mothers**		
**Maternal age (years)**		
**15–24**	89	44.40
**25–34**	78	38.80
**≥ 35**	33	16.80
**Maternal educational level**		
**Primary**	45	22.70
**Secondary**	115	58.10
**University**	38	19.20
**Marital status**		
**Married**	134	67.20
**Single**	59	29.30
**Divorced/ Widowed**	7	3.50
**Mother’s source of income**		
**Salary**	29	14.60
**Trade**	53	26.60
**Field**	32	16.10
**Spouse**	36	18.20
**None**	49	24.50
**Mother’s profession**		
**Vendor/market seller**	63	31.30
**Civil servant**	27	13.70
**Pupil/student**	32	15.90
**Housewife**	57	28.60
**Farmer**	21	10.40
**Socio-economic level**		
**Low**	21	10.70
**Medium**	56	27.90
**High**	123	61.40
**Household food security status**		
**Food security**	123	61.60
**Mild food insecurity**	69	34.80
**Moderate food insecurity**	5	2.50
**Severe food insecurity**	2	1.00
**Socio-demographics characteristics of children**		
**Child’s age (months)**		
**0–11**	183	92.00
**≥12**	17	8.00
**Child’s gender**		
**Male**	97	48.50
**Female**	103	51.50

### Prevalence of malnutrition among children under five years of age

The prevalence of malnutrition was 61.10% among children. However, Table 2 shows that the most common autonomous forms of malnutrition in children were stunting (35.00%) and overweight/obesity (34.30%), while underweight (12.80%) and wasting (8.90%) were the least common forms of malnutrition among children. Children from the mothers with low maternal age (15–25 years old) had the highest (p = 0.03) prevalence of stunting (60.00%) than those from mothers with upper maternal age (20.10%). The prevalence of underweight (45.50%) as well wasting (69.20%) were higher in children from mothers with lower education level (primary level) compared to those from mothers with higher education level. The proportion of children under twelve months having overweight/obesity was significantly higher (83.30%) than that of children aged twelve months and over (16.70%).

**Table 2:** Overall prevalence and bivariate analysis of the relation of characteristics with malnutrition among children under five years of age.

	Underweight		Wasting		Stunting		Overweight/obesity	
	% (n)	P-value	% (n)	P-value	% (n)	P-value	% (n)	P-value
General population	12.80 (22)		8.90 (13)		35.00 (55)		34.30 (60)	
**Mother’s age (years)**								
15–25	40.90 (9)	0.42	61.54 (8)	0.181	60.00 (33)	**0.03**	41.70 (25)	0.44
25–35	31.80 (7)		15.38 (2)		30.90 (17)		35.00 (21)	
≥35	27.30 (6)		23.08 (3)		09.10 (5)		23.30 (14)	
**Marital status**								
Married	81.80 (18)	0.25	76.90 (10)	0.329	58.20 (32)	0.06	66.70 (40)	0.85
Single	18.20 (4)		15.40 (2)		40.00 (22)		28.30 (17)	
Widowed/Divorced	0.00 (0)		7.70 (1)		1.80 (1)		5.00 (3)	
**Mother’s level of education**								
Primary	45.50 (10)	**0.01**	69.20 (9)	**0.00**	23.60 (13)	0.98	21.70 (13)	0.35
Secondary	31.80 (7)		30.80 (4)		61.80 (34)		51.60 (31)	
Higher	22.70 (5)		0.0 (0)		14.60 (8)		26.70 (16)	
**Socio-economic level**								
Low	4.55 (1)	0.16	15.40 (2)	0.76	9.09 (5)	0.79	8.30 (5)	0.32
Medium	40.91 (9)		30.80 (4)		29.09 (16)		21.70 (13)	
High	54.54 (12)		53.80 (7)		61.82 (34)		70.00 (42)	
**Child’s age (months)**								
0–11	95.50 (21)	0.63	100.00 (13)	0.39	94.50 (52)	0.88	83.30 (50)	**0.00**
≥12	4.50 (1)		0.00 (0)		5.50 (3)		16.70 (10)	
**Child’s gender**								
Male	45.50 (10)	0.86	23.10 (3)	0.11	50.90 (28)	0.19	51.70 (31)	0.25
Female	54.50 (12)		76.90 (10)		49.10 (27)		48.30 (29)	
**Mother’s profession**								
Vendor/market seller	36.36 (8)	0.88	15.38 (2)	0.48	32.73 (18)	0.06	35.00 (21)	0.07
Civil servant	18.18 (4)		0.00 (0)		10.91 (6)		23.33 (14)	
Pupil/student	13.64 (3)		30.77 (4)		25.45 (14)		15.00 (9)	
Housewife	18.18 (4)		38.47 (5)		27.27 (15)		21.67 (13)	
Farmer	13.64 (3)		15.38 (2)		3.64 (2)		5.00 (3)	
**Household food security status**								
Food security	54.50 (12)	0.47	53.80 (7)	0.77	65.50 (36)	0.20	68.33 (41)	0.18
Food insecurity	45.50 (10)		46.20 (6)		34.50 (19)		31.67 (19)	

### Prevalence of coexisting forms of malnutrition among children under five years of age

The prevalence CFM was 26.40% among the children. As shown in Table 3, the most prevalent coexisting form of malnutrition in children was the coexistence of stunting with overweight/obesity (14.80%) following by the coexistence of underweight with stunting (7.80%), while the coexistence of underweight with wasting was the least common form (4.20%). Children from the mothers with higher maternal age (upper 35 years old) had the highest prevalence of coexisting underweight and wasting (50.00%). The coexistence of underweight and wasting was more prevalent among children from mothers with lower education level (primary level) (83.30%) (p<0.00). The proportion of coexistence of stunting with overweight/obesity was higher (p = 0.02) in children from single mother (52.20%) than those from married mothers (43.50%).

**Table 3:** Overall prevalence and bivariate analysis of the relation of sociodemographic characteristics with of coexisting forms of malnutrition among children under five years of age.

	Coexistence of Underweight with wasting		Coexistence of Underweight with stunting		Coexistence of stunting with overweight/obesity	
	% (n)	P-value	% (n)	P-value	% (n)	P-value
General population	4.20 (6)		7.80 (12)		14.80 (23)	
**Mother’s age (years)**						
15–25	33.30 (2)	**0.02**	58.30 (7)	0.58	56.50 (13)	0.57
25–35	16.70 (1)		25.00 (3)		30.40 (7)	
≥35	50.00 (3)		16.70 (2)		13.10 (3)	
**Marital status**						
Married	83.30 (5)	0.70	75.00 (9)	0.73	43.50 (10)	**0.02**
Single	16.70 (1)		25.00 (3)		52.20 (12)	
Widowed/Divorced	0.00 (0)		0.00 (0)		4.30 (1)	
**Mother’s level of education**						
Primary	83.30 (5)	**<0.00**	16.70 (2)	0.20	34.80 (8)	0.32
Secondary	16.70 (1)		50.00 (6)		56.50 (13)	
Higher	0.00 (0)		33.30 (4)		8.70 (2)	
**Socio-economic level**						
Low	0.00 (0)	0.41	0.00 (0)	0.25	13.00 (3)	0.94
Medium	50.00 (3)		41.67 (5)		26.10 (6)	
High	50.00 (3)		58.33 (7)		60.90 (14)	
**Child’s age (months)**						
0–11	100.00 (6)	0.63	100.00 (12)	0.39	91.30 (21)	0.40
≥12	0.00 (0)		0.00 (0)		8.70 (2)	
**Child’s gender**						
Male	16.70 (1)	0.15	66.70 (8)	0.11	52.20 (12)	0.34
Female	83.30 (5)		33.30 (4)		47.80 (11)	
**Mother’s profession**						
Vendor/market seller	33.30 (2)	0.50	41.66 (5)	0.52	34.78 (8)	0.43
Civil servant	0.00 (0)		16.67 (2)		13.05 (3)	
Pupil/student	16.70 (1)		25.00 (3)		17.39 (4)	
Housewife	16.70 (1)		16.67 (2)		34.78 (8)	
Farmer	33.30 (2)		0.00 (0)		0.00 (0)	
**Household food security status**						
Food security	50.00 (3)	0.69	58.30 (7)	0.95	69.60 (16)	0.25
Food insecurity	50.00 (3)		41.70 (5)		30.40 (7)	

### Associated Risk factors for different forms of malnutrition among children under five years of age

Table 4 shows the different Odd ratios for risk factors associated to the coexisting forms of malnutrition among children. Children whose mothers had lower maternal age (15–24 years old) were at greater risk of developing stunting (OR = 3.21, 95% CI: 1.08–9.55) compared to those whose mother had upper maternal age. In addition, children whose mothers had a primary education were at greater risk of developing underweight (OR = 3.33, 95% CI: 0.34–4.34) and wasting (OR = 11.35, 95% CI: 3.20–40.16) than those whose mothers had higher education level. Children whose mothers were civil servants were at greater risk of developing overweight/obesity (OR = 2.70, 95% CI: 1.04–7.00) compared to children whose mothers had another profession.

**Table 4:** Logistic regression analysis to determine associated risk factors of malnutrition among children under five years of age.

	Underweight		Wasting		Stunting		Overweight/obesity	
	OR (95% IC)	P value	OR (95% IC)	P value	OR (95% IC)	P value	OR (95% IC)	P value
**Mother’s age**								
≥ 35 years	1		1		1		1	
25–34 years	0.46 (0.14–1.53)	0.20	0.30 (0.06–1.51)	0.14	1.53 (0.49–4.78)	0.45	0.57 (0.24–1.36)	0.21
15–24 years	0.56 (0.18–1.74)	0.32	1.48 (0.34–6.38)	0.59	3.21 (1.08–9.55)	**0.03**	0.67 (0.28–1.56)	0.35
**Maternal Marital status**								
Single	1		1		1		1	
Married	2.06 (0.66–6.45)	0.21	2.14 (0.44–10.23)	0.33	0.44 (0.21–0.91)	0.02	0.91 (0.45–1.84)	0.79
Widowed/Divorced	NA		6.50 (0.44–94.07)	0.17	0.26 (0.02–2.52)	0.24	1.36 (0.27–6.83)	0.70
**Mother’s level of education**								
Secondary/higher	1		1		1		1	
Primary	3.33 (1.31–8.44)	**0.00**	11.35 (3.20–40.16)	**<0.00**	1.00 (0.46–2.17)	0.98	0.85 (0.40–1.81)	0.69
**Mother’s profession**								
Vendor/market seller	1		1		1		1	
Civil servant	1.22 (0.34–4.34)	0.75	NA		2.19 (0.64–7.41)	0.20	2.70 (1.04–7.00)	**0.04**
Pupil/student	0.69 (0.17–2.74)	0.60	1.84 (0.38–8.91)	0.44	2.55 (0.99–6.55)	0.05	0.91 (0.34–2.40)	0.85
Housewife	0.69 (0.19–2.34)	0.54	1.80 (0.42–7.72)	0.42	1.43 (0.61–3.35)	0.40	0.98 (0.41–2.28)	0.98
**Household food security status**								
Food security	1		1		1		1	
Food insecurity	1.38 (0.56–3.41)	0.48	1.17 (0.37–3.69)	0.77	0.64 (0.32–1.24)	0.20	0.64 (0.33–1.23)	0.18

NA: Not applicable

### Associated risk factors for the coexistence forms of malnutrition among children under five years of age

Table 5 shows the different Odd ratios for risk factors associated with the coexisting forms of malnutrition among children. Children whose mothers had lower maternal age (15–24 years old) and children whose mothers had a primary education were at greater risk of coexisting underweight with wasting (OR = 23.33, 95% CI: 2.60–208.44) compared respectively to those whose mothers had upper maternal age and secondary education level. In addition, children whose mothers were single had greater risk of coexisting stunting with overweight/obesity (OR = 0.28, 95% CI: (0.11–0.73) than whose mothers were married.

**Table 5:** Logistic regression analysis to determine associated risk factors of coexisting forms of malnutrition among children under five years of age.

	Coexistence of Underweight with wasting OR (95% IC)	P value	Coexistence of Underweight with stunting OR (95% IC)	P value	Coexistence of stunting with overweight/obesity OR (95% IC)	P value
**Mother’s age**						
≥ 35 years	1		1		1	
25–34 years	0.16 (0.02–1.10)	0.06	1.16 (0.22–6.05)	0.85	1.56 (0.40–6.05)	0.51
15–24 years	0.09 (0.00–0.99)	0.05	0.56 (0.08–3.60)	0.54	0.96 (0.22–4.07)	0.95
**Marital status**						
Single	1		1		1	
Married	2.17 (0.24–19.21)	0.48	1.32 (0.34–5.14)	0.68	0.28 (0.11–0.73)	**0.00**
Widowed/Divorced	NA		NA		0.68 (0.07–6.78)	0.74
**Mother’s level of education**						
Secondary/higher	1		1		1	
Primary	23.33 (2.60–208.44)	**0.00**	0.63 (0.13–3.04)	0.57	1.89 (0.73–4.90)	0.18
**Mother’s profession**						
Vendor/market seller	1		1		1	
Civil servant	NA		NA		2.40 (0.53–10.87)	0.25
Pupil/student	0.55 (0.05–5.20)	0.60	1.92 (0.39–9.27)	0.44	1.45 (0.38–5.46)	0.57
Housewife	0.42 (0.04–3.99)	0.45	0.84 (0.14–4.84)	0.84	1.75 (0.56–5.44)	0.33
**Household food security status**						
Food security	1		1		1	
Food insecurity	1.38 (0.27–7.11)	0.69	1.03 (0.31–3.41)	0.95	0.57 (0.22–1.49)	0.25

NA: Not applicable

## Discussion

In the present cross-sectional study, we assessed the prevalence and risk factors of coexisting forms of malnutrition (CFM) among a cohort of children in a rural area of Cameroon. We observed a very high prevalence of single forms of malnutrition which were 12.80%, 8.90%, 35.0%, and 34.30% respectively for underweight (weight for age), wasting (weight for height), stunting (height for age), and obesity (BMI for age). The prevalence findings are consistent with those found in other rural areas accross the country [14]. However, these prevalences are higher than those of stunting (22.80%-29.90%), wasting (1.60%–5.10%) and undernutrition (9.58%-14.34%) found from 2010 to 2019 across the country [23]. This confirms the fact that malnutrition remains a public health concern in Cameroon, particularly among children under five years age in rural areas. We further determined the prevalence of CFM among children. We found three major types: the coexistence of underweight with wasting (4.20%), the coexistence of underweight with stunting (7.80%) and the coexistence of stunting with overweight (14.80%). The prevalence of coexisting of underweight with wasting and the coexisting of underweight with stunting are less than those found in Somalia (20.00% and 29.00%) [24] and Tanzania (21.00% and 33.00%) [25]. Stunting mostly refers to chronic malnutrition, wasting to acute malnutrition, underweight encompasses both two forms [26]. The coexistence of stunting with underweight as well as wasting with underweight among children could be because of persisting nutritional deficiency due to inadequate feeding practice since infancy or early childhood [27].

The coexisting of stunting with overweight /obesity was the most prevalent form of CFM among children. The findings prevalence is higher compared to those found in most of African countries. In fact, in studies conducted in Ethiopia [28], Ghana [29], Kenya [30] and Libya [31] found prevalences of 1.99%, 2.80%, 1.00% and 5.06% respectively. The possible explanation of higher prevalence of this type of CFM may be the fact that children are fed with high-calories diet leading to weight gain, but deficient in micronutrients such as zinc affecting child’s growth. Taking all of these in account we suggest that most children in rural area Cameroon should be fed with food containing important nutrients such as iron, iodine, vitamin A, and zinc, specific proteins and long-chain polyunsaturated fatty acids (n-3 and n-6 PUFA) which are beneficial for a child’s growth. On the contrary, children are fed with high energy-rich food such as starch which leads to overweight. However, further studies are needed to evaluate the infant’s feeding practices which would provide information about the food groups and nutrients that have beneficial or adverse effects on a child’s nutritional status. This is one of the limitations of the current study.

In this study we also investigate the associations between maternal infant characteristics and CFM among children. We found that children from mothers with lower maternal age (15–24 years old), and lower education level were more likely to be concurrently underweight and wasting. These results are consistent with those reported by Fernald & Neufeld [32] among children 24–72 months of age in rural area of Mexico. Furthermore, we observed that children born to single mothers were more likely to be concurrently stunted with overweight/obesity. These findings highlight the direct impact of maternal socio-characteristics on child’s nutritional status in the population. Furthermore, it has been noted that each CFM is associated with specific maternal factors.

The findings of this study highlight both the persistence of high rates of stunting, underweight, wasting and overweight and the coexistence of those different forms of malnutrition among children under five in rural areas of the country. None of the studies have previously reported the coexistence of different forms of malnutrition among children under five in the country. This may explain why malnutrition rates in Cameroon remain high, although numerous nutrition policies and programs are implemented. This may be because nutritional policies and programs don’t take to account both single and coexistence forms of malnutrition among children. Furthermore, the mother’s social status should be taken to account in nutritional policies to fight child malnutrition in rural areas of country. It also important to take into consideration the mother’s nutritional status which may be associated with malnutrition in children. This is another important point that need to be investigated.

### Strengths and limitations of the study

To the best of our knowledge, this is the first study conducted in Cameroon, which has shown evidence concerning the prevalence of coexisting forms of malnutrition in children and identify the factors associated with this coexisting form of malnutrition. The resulting findings can be used to better address remaining questions about the double burden of malnutrition in children in Cameroon. However, the cross-sectional design and the small sample size of the study limit the generalization of the available evidence.

## Conclusion

In conclusion, the findings of this study provided evidence on the co-existence of different forms of malnutrition among children under five years of age in a rural area of Cameroon. The most prevalent form was the coexistence of stunting with overweight. The significant factors associated with coexisting forms of malnutrition among children included lower maternal age, lower education level and unmarried status. These findings provide useful insight into the prevalence and characteristics of coexisting forms on malnutrition in children and would guide future research, policies, and programs to manage all forms of malnutrition among children particular in rural area of Cameroon.
